# Spray Nozzles, Pressures, Additives and Stirring Time on Viability and Pathogenicity of Entomopathogenic Nematodes (Nematoda: Rhabditida) for Greenhouses

**DOI:** 10.1371/journal.pone.0065759

**Published:** 2013-06-06

**Authors:** Grazielle Furtado Moreira, Elder Simões de Paula Batista, Henrique Borges Neves Campos, Raphael Emilio Lemos, Marcelo da Costa Ferreira

**Affiliations:** Department of Fitossanidade, Universidade Estadual Paulista (UNESP), Jaboticabal Campus, Jaboticabal, São Paulo, Brazil; Dowling College, United States of America

## Abstract

The objective of this study was to evaluate different strategies for the application of entomopathogenic nematodes (EPN). Three different models of spray nozzles with air induction (AI 11003, TTI 11003 and AD-IA 11004), three spray pressures (207, 413 and 720 kPa), four different additives for tank mixtures (cane molasses, mineral oil, vegetable oil and glycerin) and the influence of tank mixture stirring time were all evaluated for their effect on EPN (*Steinernema feltiae*) viability and pathogenicity. The different nozzles, at pressures of up to 620 kPa, were found to be compatible with *S. feltiae*. Vegetable oil, mineral oil and molasses were found to be compatible adjuvants for *S. feltiae*, and stirring in a motorized backpack sprayer for 30 minutes did not impact the viability or pathogenicity of this nematode. Appropriate techniques for the application of nematodes with backpack sprayers are discussed.

## Introduction

Entomopathogenic nematodes (EPN) belonging to the genera *Steinernema* and *Heterorhabditis* (Rhabditida: Steinernematidae, Heterorhabditidae) have been used effectively in biological control of soil pests - especially Lepidoptera, Coleoptera and Diptera [1]. Subsequently, EPN have become another important tool for integrated pest management programs (IPM). These organisms are generally resistant to many crop protection products. They have synergistic action with other entomopathogenic agents and the ability to disperse in the environment, while posing no risk to plants and other animals, including humans [2]. Controlled environments, such as cultivated crops, benefit from the effects of these nematodes because of their bioecological characteristics. According to Grewal [3], the EPN can be applied using conventional spraying systems and irrigation systems. However, survival after application (due to environmental factors and/or the application process) is a major obstacle in expanding the use of these agents as biopesticides. To achieve a satisfactory pest control using entomopathogens, it is necessary for the application technique to be effective, because some application factors, such as the process of droplet formation, the size and type of nozzle, spray pressure and pumping system, can reduce the viability of these organisms [4].

The choice and appropriate use of spray nozzles is essential for the correct application of the crop protection product on the target [5]. The characteristics of the target pest determine the preferred droplet size, and droplet size is determined by multiple factors, including the model of the spray nozzle, the spray pressure used, the opening angle and the filter mesh [6]. According to Cunha et al. [7] the use of larger diameter drops (>200 µm) are used in control of target pests in the soil. Furthermore, the same authors discussed that the use of an adjuvant in the spray mixture could reduce evaporation, increasing the longevity of a droplet. Because the EPN are sensitive to desiccation, adjuvants might assist in the application of EPN in the field, provided they are compatible with the EPN.

Stirring the tank mixture during the pulverization process ensures homogeneity, which facilitates the application of entomopathogens due a better distribution of the active ingredient. However, friction with the inner parts of the hydraulic circuit can cause physical damage to entomopathogens [8]. According to Fife et al. [9] the cuticle structure of the infective juvenile (IJ), which can change in different species, can respond differently to hydrodynamic stress. Additionally, there might be an increase in tank mixture temperature throughout this process, which may reduce viability of the nematodes. Fife et al. [10] considered thermal influences during pump recirculation to be more detrimental to EPN than mechanical stress Therefore, pumps with lower capacity, such as diaphragm or roller pumps, seem to be better for biopesticides. According to Lara et al. [11], in one hour of agitation, the tank mixture temperature can increase from 22 to 43°C in a tank with a centrifugal pump, and from 22 to 27°C in a tank with diaphragm or peristaltic pumps. Therefore, temperature and stirring of the tank mixture are also important factors to consider in the application of EPN.

The nematode *S. feltiae* has been found to control various pests, which cause serious damages [12], [13], [14]. Conventionally, the wax moth [*Galleria mellonella* (Lepidoptera: Pyralidae)] is the host used around the world to evaluate the viability and pathogenicity of EPN because it is susceptible to a wide range of nematode species [15]. The nematodes of the genus *Steinernema* are normally larger than the *Heterorhabditis* species. *S. feltiae*, for example, has a medium size of 849 µm, whereas the largest *Heterorhabditis* species, *H. megidis*, has a medium size of 768 µm [16]. According to Fife [17], the size of the nematodes relates to their viability during an application process. Therefore, *S. feltiae* is a good species to use to test the influence of the application process on nematode survival.

Because EPN can be used to control soil pests in greenhouses, the objective of our study was to examine a simple way of using this biopesticide in greenhouses by evaluating the impact of several application techniques on the viability and pathogenicity of EPN. The following factors were evaluated for their impact on the EPN *S. feltiae*: models of spray nozzles with air induction, spray pressure, the use of adjuvants in the tank mixture and stirring time.

## Materials and Methods

### Obtaining the entomopathogenic nematode and preparing the tank mixture

*S. feltiae* was provided by the Bank of Entomopathogens, Laboratório de Patologia de Insetos, Federal University of Lavras (UFLA), Lavras, Brazil. To obtain the IJ, we used 30 larvae of *G. mellonella*, reared according to the methodology described by Dutky et al. [18]. An artificial diet was used [19]. These caterpillars were transferred to Petri dishes (9 cm diameter) containing two sheets of filter paper, and later an aqueous suspension of 2 mL with nematodes was added. The dishes were kept in a Biochemical Oxygen Demand incubator for 72 hours (Temperature: 24±2°C, RH 70%±10% and photoperiod of 14 hours). After infection, the larvae were transferred to a dry chamber [20], where they were kept for four days. After this time, the larvae were transferred to White traps [21] for collection of the IJ.

For preparation of sprays composed of water and nematodes, the IJ were quantified in one mL of aqueous suspension, using a stereoscopic microscope. The concentration used was 75 000 IJ/L of spray, as suggested by Garcia et al. [8].

### Influence of spray nozzles and pressures

The experiments were performed in 2010 and repeated in 2011 and comprised 9 treatments, with three nozzle models at three pressures, consisting of three replicates per treatment. The spray nozzles were: AI 11003 (air induction nozzle model by Teejet, producing coarse droplets), TTI 11003 (deflector air induction nozzle model by Teejet, producing extra coarse droplets) and AD-IA 11004 (air induction nozzle model by Magno Jet, producing coarse droplets). All nozzles were flat-fan air induction, which provide coarse to extremely coarse droplets, ideal for targets in the soil. The nozzles were each operated at pressures of 207, 413 and 620 kPa, with a filter size of 100 mesh. The tank mixture of tap water and IJ from the nematode *S. feltiae* was added to a stainless steel tank with a volume capacity of five liters and was pressurized with compressed air. Each nozzle was placed individually in a spray boom, and the sprayed liquid was collected for 15 seconds in an Erlenmeyer flask. Tap water and IJ mixture without pressurization was used as a control sample.

To assess viability, the percentage of living and dead nematodes was determined by withdrawing one mL from each treatment and making a random count of 100 IJ. To evaluate pathogenicity, 200 mL was removed from the suspension of each trial and pipetted into a Petri dish with two filter sheets containing five *G. mellonella* larvae, as suggested by Andaló et al. [22]. After five days, dead larvae were quantified by observing the typical symptoms of infection by nematodes of the genus *Steinernema*.

The data concerning the viability and pathogenicity of *S. feltiae* were subjected to normality test (Shapiro-Wilk), variance analysis and averages were compared through Tukey test at 1% probability, with data transformation √ *x*+0.5, when necessary.

### Compatibility between *Steinernema feltiae* and adjuvants

To evaluate the compatibility of *S. feltiae* and adjuvants, different tank mixtures were prepared, containing the nematode and concentration of 1% of the volume of sugar cane molasses (agro-industrial by-product of sugar), vegetable oil (Veget'oil by Oxiquímica Agrociência, Ltda.), mineral oil (Attach by BASF), or a glycerin-based treatment (GL-1 by Pulsfog Pulverizadores Ltda.). Each treatment was placed in a stainless steel tank with a volume capacity of five liters and pressurized with compressed air, consisting of three replicates per treatment. For the mixing, the TTI 11003 nozzle was used, at a pressure of 413 kPa, placed at the spray bar. The pulverized spray was collected for 15 seconds in an Erlenmeyer flask. Tap water and IJ mixture without adjuvants was used as a control sample.

The evaluation of the viability and pathogenicity of *S. feltiae* in each treatment was performed with the percentage of living and dead nematodes and the number of dead *G. mellonella* larvae, respectively, as described in the previous experiment [22]. The data were subjected to normality tests (Shapiro-Wilk), and variance analyses and averages were compared through Tukey test at 5% probability, with data transformation √ *x*+0.5.

### Influence of stirring time of spray on *Steinernema feltiae*

To perform this experiment, a seven-liter tank mixture containing water and nematode was prepared. To stir the tank mixture, a motorized backpack sprayer (Yamaha LS-937) was used with two-stroke 23 cc engine, a maximum pressure of 2068 kPa and a tank capacity of 25 L. The sprayer worked at maximum return of liquid flow. Three 200 mL aliquots were taken from the tank at four different stirring times: 0, 10, 20 and 30 minutes, consisting of three replicates per treatment. The temperature of the spray liquid at each time was measured using a thermometer. From the collected aliquots, the viability and pathogenicity of *S. feltiae* was assessed as described above. The data were subjected to normality tests (Shapiro-Wilk), and variance analyses and averages were compared through Tukey test at 1% probability, with data transformation √ *x*+0.5, when necessary.

## Results

### Influence of spray nozzles and pressures

There was no significant difference in the viability of *S. feltiae* with the three different nozzles (Tables 1 and 2). In the first experiment with the nozzles AI 11003, TTI 11003, and AD-IA 11004, there was no influence on the viability of *S. feltiae* (Table 1) at the three evaluated pressures. However, in the second experiment, the nozzle TTI 11003 showed a significant difference in the viability of nematodes at a pressure of 207 kPa (Table 2).

**Table 1:** Viability (%) and pathogenicity (%) of infective juveniles of *Steinernema feltiae* subjected to different nozzles at different pressures in 2010.

Pressure (kPa)	Viability (%)			Pathogenicity (%)*		
	AI 11003	TTI 11003	AD-IA 11004	AI 11003	TTI 11003	AD-IA 11004
207	96.66 Aa	95.33 Aa	96.33 Aa	93.33 Aa	100.00 Aa	93.33 Aa
413	94.33 Aa	97.00 Aa	95.66 Aa	86.66 Aa	100.00 Aa	93.33 Aa
620	97.00 Aa	95.66 Aa	96.66 Aa	100.00 Aa	100.00 Aa	93.33 Aa
Control Sample^1^	98.00 A	98.00 A	98.00 A	100.00 A	100.00 A	100.00 A
CV (%)		1.2		6.28		

1 Spray not subjected to pressurization; averages followed by the same uppercase letters in columns and lowercase letters in lines are not different according to Tukey test results (P≤0.01).

* Original pathogenicity data were transformed into √ *x*+0.5.

**Table 2:** Viability (%) and pathogenicity (%) of infective juveniles of *Steinernema feltiae* subjected to different nozzles at different pressures in 2011.

Pressure (kPa)	Viability (%)			Pathogenicity (%)*		
	AI 11003	TTI 11003	AD-IA 11004	AI 11003	TTI 11003	AD-IA 11004
207	94.33 Aa	92.00 Ba	93.00 Aa	86.66 Aa	100.00 Aa	80.00 Aa
413	96.66 Aa	93.00 ABa	95.33 Aa	100.00 Aa	100.00 Aa	100.00 Aa
620	94.66 Aa	94.00 ABa	94.33 Aa	100.00 Aa	86.66 Aa	100.00 Aa
Control Sample^1^	97.66 A	97.66 A	97.66 A	100.00 A	100.00 A	100.00 A
CV (%)		1.79		7.29		

1 Spray not subjected to pressurization; averages followed by the same uppercase letters in columns and lowercase letters in lines are not different according to Tukey test results (P≤0.01).

* Original pathogenicity data were transformed into √ *x*+0.5.

The results related to pathogenicity in both experiments show that all of the nozzles used at the tested pressures did not affect the pathogenicity of *S. feltiae*. The mortality in larvae of *G. mellonella* was greater than 80%, not differing significantly from the control treatment that had not gone through the pressurization process (Table 1 and 2).

### Compatibility between *Steinernema feltiae* and different adjuvants

**Figure 1:**
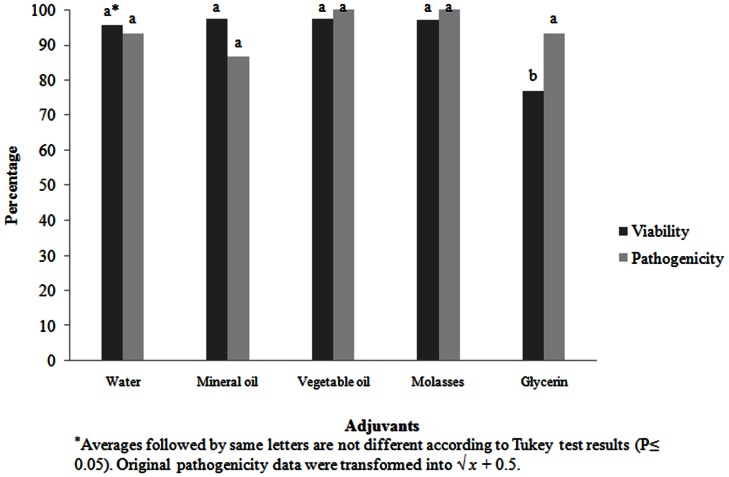
Compatibility of spray with different adjuvants on viability and pathogenicity of *Steinernema feltiae*.

From the various adjuvants used, vegetable oil, mineral oil and molasses were shown to be compatible with *S. feltiae*, with the IJ viability of 97%, not differing significantly from the control treatment (95%). The lowest viability (77%) was obtained in the tank mixture containing glycerin-based treatments, which differed significantly from the other ones. Concerning pathogenicity, however, there was no significant difference between treatments, and the glycerin-based treatments had a 93% mortality rate with the *G. mellonella* larvae (Figure 1).

### Influence of stirring time of the spray on the viability and pathogenicity of *Steinernema feltiae*

The stirring time of the tested spray (0, 10, 20 and 30 minutes) did not affect the viability and pathogenicity of *S. feltiae*. The initial temperature of the spray (time 0) during the stirring was 26°C and peaked at 30°C sampled at the last moment, after stirring for 30 minutes (Table 3).

**Table 3:** Viability and pathogenicity of *Steinernema feltiae* after spray had been exposed to motorized knapsack sprayer at different stirring times.

Time (min)	Temperature (°C)	Viability (%)	Pathogenicity (%)*
0	26	97.66^1^ a	100.00 a
10	28	96.00 a	100.00 a
20	29	96.33 a	66.66 a
30	30	96.00 a	86.66 a
CV (%)		1.1	11.82

1 Averages followed by same letters are not different according to Tukey test results (P≤0.01).

* Original pathogenicity data were transformed into √ *x*+0.5.

## Discussion

We sought to establish a simple way of using NEP in greenhouses. We observed that different flat-fan air induction spray nozzles models, spray pressure (207 kPa to 620 kPa), adjuvants and the stirring time using a motorized backpack sprayer did not affect the viability and pathogenicity of EPN.

Regarding the choice of air induction spray nozzles, all have shown compatibility with *S. feltiae* with values of viability and pathogenicity greater than 80%. These results show that air induction nozzles, commonly recommended in applying pre-emergent herbicides, are suitable for the application of EPN, mainly due to the droplet size that they provide. According to Matthews [23], nozzles with air induction - such as the three tested nozzles - produce large droplets. Thus, air induction nozzles are recommended for targets that are horizontal, such as soil pests. According to Ferraz [2], the EPN are quite sensitive to desiccation. Therefore, the use of large droplets is necessary to optimize the application of nematodes in the soil. Additionally, small droplets have a lower final velocity [24], increasing the time taken for deposition on the target and therefore making them more susceptible to evaporation and change of trajectory.

Garcia et al. [25], evaluating the influence of different nozzles on the viability of the nematodes *H. indica* and *Steinernema* sp., found a significant reduction in viability (25%) for the air induction nozzle AI 110015, with a mesh filter size of 100 mesh and 200 kPa pressure. According to those authors, the use of the filter interfered with the viability of these nematodes. This result differs from our present study in which 100 mesh was used for all the evaluated nozzles. However, those authors also noted that the concentration of nematodes in the sprayed liquid is reduced significantly using the 100 mesh filter if compared with filters of 25 and 50 mesh and no use of a filter. Therefore, the use of filters in the application of nematodes must be made carefully.

Using the nozzles XR8001VS flat fan, TXA8001VK hollow cone and FL5-VS full cone to evaluate the viability of *H. bacteriophora*, *H. megidis*, *Steinernema carpocapsae* and *S. glaseri* in different volumetric flow rates, Fife et al. [17] found difference among species but found survival rates as high as 85%. According to these authors, the size of the nematodes can influence their viability. They recommend cone nozzles over fan nozzles because the latter can cause damage to the EPN, although no pathogenicity tests were performed.

The results obtained in this study, with pressures of 207 to 620 kPa, corroborate other research indicating that pressurization of the spray has low interference with the viability and pathogenicity of EPN. According to Grewal [3], entomopathogenic nematodes tolerate pressures up to 2070 kPa. Fife et al. [26], using different pressures (1283 kPa to 10690 kPa) to evaluate the survival of the nematodes *S. carpocapsae*, *H. bacteriophora* and *H. megidis* in suspensions with different age, found difference among the species in tolerance of high pressures, recommending a maximum operate pressure of 1380 kPa. The same authors observed that the age of nematode suspension (maximum 3 weeks) did not influence the tolerance of pressures effects. Garcia et al. [8] found over 80% viability in *S. glaseri* using pressures up to 1379 kPa during spraying. Nilsson and Gripwall [4], having sprayed the nematode *S. feltiae* at different pressures, also found no significant difference in their viability at pressures up to 2000 kPa. Currently, though, pressures above 1000 kPa have not been commonly used, and a range between 200 and 600 kPa is ideal. Spraying at higher pressures results in a finer and more airborne mist, increased wear on the nozzle material and more energy consumption during the spraying operation. Therefore, spraying at lower pressure results in increased environmental safety and conservation of resources and equipment.

As already reported in several studies, the use of adjuvants improves the characteristics of the spray, also changing the surface tension of the sprayed liquid, which consequently slows down the evaporation process [27]. Because nematodes are sensitive to desiccation, the use of adjuvants - preferably from natural ingredients - such as sugar cane molasses and vegetable oil, may be favorable. Compatibility of the EPN with some adjuvants (as shown in this study) indicates that adjuvants are desirable to use during the application of EPN. Petroleum-based adjuvants, such as glycerin and mineral oil, can reach groundwater, causing environmental contamination. In Brazil, soybean oil produced for agricultural spraying has been widely used due to its low cost and adequate availability [28]. Sugar cane molasses (an industrial by-product obtained from sugar) is easily obtained in sugarcane-producing regions and has been used in applications with ultra low volume (ULV) in Brazil.

The continuous stirring of the spray improves the application of EPN because it allows the concentration of IJ by volume (or dilution) to be more homogeneous. In addition to ensuring good uniformity of the spray with the stirring process, the motorized backpack sprayer demonstrated no effect on viability and pathogenicity of *S. feltiae*. The motorized stirrer most likely does not cause physical damage to nematodes. After 30 minutes of stirring, the temperature of the spray mixture reaches 30°C and can therefore be used for the application of EPN in greenhouses. According to Grewal [3], in the application equipment for nematodes, temperatures above 30°C should be avoided.

Nilsson and Gripwall [4], using a high pressure spray pump piston, found a reduction of approximately 10% in the viability of *S. feltiae* sprayed after 20 minutes of stirring. The temperature of the spray in this sprayer increased from 19 to 23°C. According to these authors, the reduction in viability can be explained by the mechanical stress, the mesh used (50 mesh) or by the increase in temperature. The pressurization system used achieves higher values compared to backpack sprayer used in present study. Using a manual backpack sprayer with no stirring, these authors found no reduction in the viability of nematodes.

Fife et al. [10] used different valves (full ball valve, reduced ball valve, diaphragm, globe and needle) and pump types (centrifugal, diaphragm and roller) to evaluate possible damages on the nematode *H. bacteriophora* on a single passage through these valves and pumps. They observed no physical damage on *H. bacteriophora* when using the different valves and pumps, observing viability rates of up to 97% (untreated control: 96%). These authors also evaluated changes in the temperatures (using just water) for different pumps and they observed an increase of 8.5, 11.2 and 33.6°C in centrifugal, diaphragm and roller pump, respectively, after 2 hours, suggesting that thermal influences may be more detrimental to EPN than mechanical stress.

Our current studies indicate that EPN can tolerate different stress mechanisms and suggest that alternative adjuvants should be used, such as sugar cane molasses and vegetable oil; thus more investigations evaluating different adjuvants would be interesting to improve the field application.

## Conclusion

Based on the results found in this study (i.e., no reduction in EPN viability or pathogenicity), using spray nozzles with air induction and pressures near 400 kPa ensures a good application of entomopathogenic nematodes using motorized backpack sprayers in a greenhouse, and the use of adjuvants in the spray mixture with this entomopathogen may be favorable to the spraying process.
